# Effect of sequential C‐terminal tryptophans on green fluorescent protein fluorescence

**DOI:** 10.1002/2211-5463.12445

**Published:** 2018-05-29

**Authors:** Shirou Tsuchida, Takumi Kanashiki, Shuhei Izumiya, Takuya Ichikawa, Ryusuke Kurosawa, Naoya Hamaue, Takashi Aoki

**Affiliations:** ^1^ Department of Molecular Biosciences School of Pharmaceutical Sciences Health Sciences University of Hokkaido Ishikari‐Tobetsu Japan

**Keywords:** C terminus, cloning vector, *Escherichia coli*, green fluorescent protein, tryptophan

## Abstract

The effect of the addition of sequential C‐terminal tryptophan residues on the fluorescence intensity of GFP was investigated. Tandem repeats of six tryptophan residues markedly decreased fluorescence intensity. This phenomenon is likely to occur because of the inhibition of GFP folding, resulting in insolubility. Exploiting this phenomenon, we constructed a cloning vector that facilitates the identification of recombinant colonies of *Escherichia coli* by the activation of GFP.

**Abbreviations**
CBB: Coomassie Brilliant Blue
GFP: green fluorescent protein

Green fluorescent protein (GFP) fluoresces only when irradiated by UV light. It was isolated and purified from the jellyfish *Aequorea* by Shimomura *et al*. in 1962 1. Since then, it has proven indispensable in biochemistry studies. Commonly, GFP is utilized as a fusion protein for the visualization of intracellular localization 2, 3 and as an indicator of gene activation, in so‐called ‘reporter assays’ 4. The fluorescence intensity of GFP varies with the type of peptide fused to the C terminus, and we have previously reported on the relationship between GFP fluorescence intensity and such fused polypeptides 5, 6. We found that the fluorescence intensity of GFP fused to polypeptides increases with the product of the average hydrophilicity of the fused segment multiplied by the number of fused amino acids. The ability to arbitrarily turn GFP fluorescence on or off is considered to be very useful. In this study, the effect of a small number of tryptophan (Trp) residues added to the GFP C terminus was investigated. A series of these inhibited GFP folding, resulting in insolubility and loss of fluorescence. Applying this phenomenon, we constructed a cloning plasmid vector whose fluorescence is turned on when foreign DNA is inserted into the cloning site.

## Materials and methods

### pS147PGFP as a basic vector

**Figure 1:**
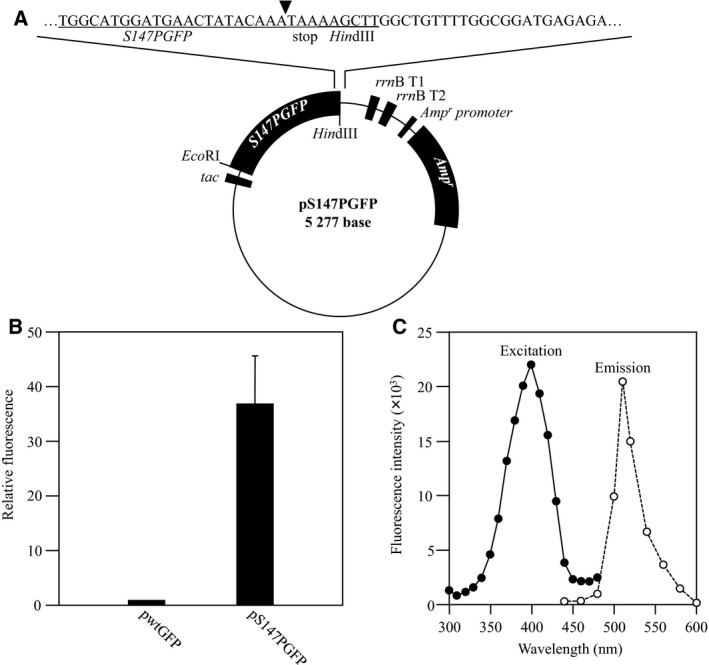
Structure of pS147PGFP (A) and fluorescence intensity of S147PGFP (B, C). (A) The Trp insertion position is indicated by a closed triangle. (B) The fluorescence intensity of *Escherichia coli* harboring pS147PGFP was measured and relative fluorescence intensity was obtained by setting the fluorescence intensity of *E. coli* harboring plasmid encoding wild‐type GFP (pwtGFP) as 1. (C) The excitation spectrum was obtained with emission set at 405 nm, and the emission spectrum was obtained with excitation set at 535 nm.

Trp‐appended GFP (pGFP‐Trp_*n*_, where *n* indicates the number of added amino acid residues) was constructed by modification of pS147PGFP 7. The plasmid map of pS147PGFP is shown in Fig. 1A. pS147PGFP was constructed by insertion of the S147PGFP gene (*S147PGFP*) between the *Eco*RI and *Hin*dIII restriction sites of a pkk223‐3 expression vector (GE Healthcare, Little Chalfont, UK). To enhance fluorescence, the 147th residue (where the methionine start codon is defined as the first residue), a serine, was replaced by proline (Fig. 1B). The peaks of excitation and emission were approximately 400 and 520 nm, respectively (Fig. 1C).

### Chemicals and instruments

Oligonucleotides for inverse PCR or site‐directed mutagenesis were synthesized by Thermo Fisher Scientific (Carlsbad, CA, USA). Inverse PCR was performed using the KOD Plus Mutagenesis Kit (Toyobo, Osaka, Japan). Site‐directed mutagenesis was performed using Pfu turbo DNA polymerase (Agilent Technologies, Santa Clara, CA, USA). Plasmids were purified from cells using the PureLink Quick Plasmid Miniprep Kit (Invitrogen, Carlsbad, CA, USA). *Dpn*I was purchased from Invitrogen. PCR was performed using an iCycler (Bio‐Rad, Hercules, CA, USA). Sequencing was performed with a 3130 genetic analyzer (Thermo Fisher Scientific). Fluorescence of colonies was evaluated using a 3UV transilluminator (UVP, Upland, CA, USA). The turbidity and fluorescence intensity of *Escherichia coli* in liquid medium were measured using a SPECTRAFluor Plus microplate reader (Tecan, Männedorf, Switzerland). Centrifugation was performed using a Centrifuge 5415R (Eppendorf, Hamburg, Germany) equipped with a F45‐24‐11 fixed‐angle rotor. SDS/PAGE gels were stained using a SeePico CBB stain kit (Benebiosis, Seoul, Korea).

### Construction of the plasmids

The pGFP‐Trp_*n*_ series was constructed by insertion of Trp codons (TGG) between the C‐terminal Lys (AAA) and the stop codon (TAA). pGFP‐Trp_1_ and pGFP‐Trp_5_ were constructed by inverse PCR using the following primers: forward, 5′‐(TGG)_*n*_ TAA AAGCTTGGCTGTTTTGG‐3′ (*n* = 1, 5) (the stop codon is underlined and the *Hin*dIII recognition site is double underlined), and reverse, 5′‐TTTGTATAGTTCATCCATGCCATGT‐3′. Inverse PCR was performed by cycling at the following temperatures: 25 cycles of 1 min at 95 °C, 30 s at 50 °C and 1 min at 68 °C. Digestion of the template plasmid and subsequent phosphorylation and ligation of the PCR product were performed according to the protocol of the KOD Plus Mutagenesis Kit. *E. coli* JM109 cells prepared by a calcium chloride method 8 were transformed with the construct. The transformant was cultured on a LB agar plate containing ampicillin (final concentration, 50 μg·mL^−1^) at 37 °C overnight (approximately 20 h). After culture, the colonies were picked and cultured in 10 mL of LB medium containing ampicillin (final concentration, 50 μg·mL^−1^). Plasmids were purified using the PureLink Quick Plasmid Miniprep Kit. Sequencing the plasmids using a 3130 genetic analyzer confirmed the correct insertion of the Trp codons. In inverse PCR, pGFP‐Trp_6_ was produced unintentionally. pGFP‐Trp_2_, pGFP‐Trp_3_ and pGFP‐Trp_4_ were constructed by site‐directed mutagenesis 9, 10 using the following primers: forward, 5′‐CTATACAAA(TGG)_*n*_ TAA AAGCTTGGC‐3′ (*n* = 2, 3, 4, 5), and reverse, 5′‐GCCAAGCTT TTA(CAA)_*n*_TTTGTATAG‐3′ (*n* = 2, 3, 4, 5). Site‐directed mutagenesis using Pfu turbo DNA polymerase was achieved by cycling at the following temperatures: 16 cycles of 1 min at 95 °C, 30 s at 45–55 °C and 13 min at 68 °C. The annealing temperature was adjusted according to the *T*_m_ value of each primer. After digestion of the template plasmid by *Dpn*I at 37 °C for 1 h, *E. coli* JM109 cells were transformed with the construct. The subsequent procedure was the same as for the construction of pGFP‐Trp_1_ and pGFP‐Trp_5_ (see above). pGFP‐Leu_*n*_ (*n* = 2, 4, 6) was constructed in the same way as pGFP‐Trp_*n*_ using the following primers: forward, 5′‐CTATACAAA(CTG or TTA)_*n*_ TAA AAGCTTGGC‐3′ (*n* = 2, 4, 6), and reverse, 5′‐GCCAAGCTT TTA(CAG or TAA)_*n*_TTTGTATAG‐3′ (*n* = 2, 4, 6). In addition, pGFP‐Gly_*n*_ (*n* = 2, 4, 6) was constructed in the same way as pGFP‐Trp_*n*_ using the following primers: forward, 5′‐CTATACAAA(GGT)_*n*_ TAA AAGCTTGGC‐3′ (*n* = 2, 4, 6), and reverse, 5′‐GCCAAGCTT TTA(ACC)_*n*_TTTGTATAG‐3′ (*n* = 2, 4, 6). pGFP‐SmaI‐Trp_*n*_ (*n* = 6, 8) was constructed as was pGFP‐Trp_*n*_ using the following primers: forward, 5′‐**CCCGGG**(TGG)_*n*_ TAA AAGCTTGGCTGTTTGG‐3′ (*n* = 6, 8) (the *Sma*I recognition site is shown in bold), and reverse, 5′‐TTTGTATAGTTCATCCATGCCATGT‐3′.

### Measurement of fluorescence intensity

*Escherichia coli* harboring pGFP‐Trp_*n*_ were cultured in 10 mL of LB medium containing ampicillin (final concentration, 50 μg·mL^−1^) at 37 °C overnight (approximately 20 h) and cells were harvested from 1 mL of the medium. After washing with PBS (137 mm NaCl, 2.7 mm KCl, 1.5 mm KH_2_PO_4_, 8.1 mm NaHPO_4_, pH 7.4), the cells were resuspended in 1 mL of PBS. Turbidity of the suspensions was measured at 620 nm and fluorescence intensity was measured at an excitation of 405 nm and emission of 535 nm.

### Electrophoresis

The cells harvested from 10 mL LB medium after the culture (see above) were disrupted by sonication in 1 mL of PBS. The sonicate was centrifuged (13 362 ***g***, for 10 min at 4 °C) and 10 μL of supernatant or pellet mixed with 10 μL of sample buffer [125 mm Tris/HCl, pH 6.8, 10% (v/v) 2‐mercaptoethanol, 4% (w/v) SDS, 10% (w/v) sucrose, 0.01% (w/v) bromophenol blue]. Samples were subjected to SDS/PAGE ether with or without boiling in the presence of mercaptoethanol. The polyacrylamide concentrations of the stacking and separation gels were 6% and 12%, respectively. Electrophoresis was performed at a constant voltage of 150 V with running buffer of 25 mm Tris, 192 mm glycine and 0.1% SDS. The fluorescing band of GFP in non‐boiled samples was observed under UV‐A (365 nm) irradiation using a transilluminator, and the gel then stained with Coomassie Brilliant Blue (CBB). In the case of boiled samples, gels were immediately stained with CBB after electrophoresis. Densitometry data of the bands were obtained using imagej software 11.

## Results and Discussion

### Effect of C‐terminal Trps on GFP fluorescence

**Figure 2:**
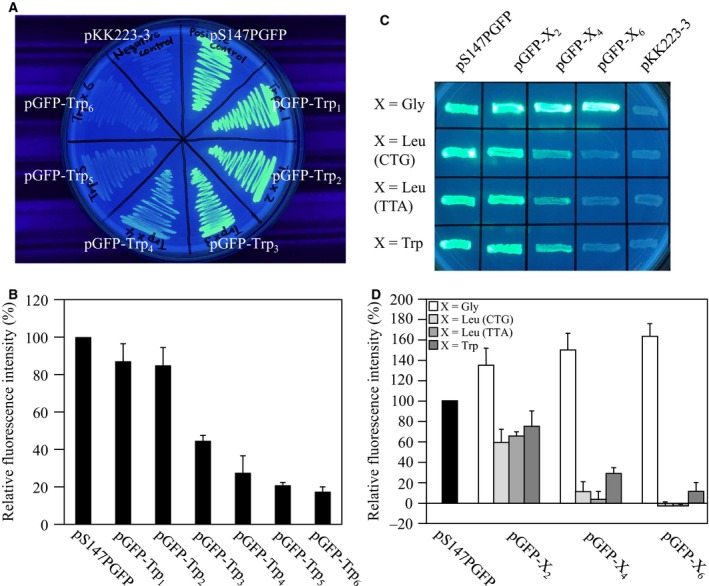
(A) Appearance of *Escherichia coli* harboring pGFP‐Trp_*n*_. (B) Fluorescence intensity of *E. coli* harboring pGFP‐Trp_*n*_. (C) Appearance of *E. coli* harboring pGFP‐Leu_*n*_ and pGFP‐Gly_*n*_. (D) Fluorescence intensity of *E. coli* harboring pGFP‐Leu_*n*_ and pGFP‐Gly_*n*_.

*Escherichia coli* colonies harboring pGFP‐Trp_1_, pGFP‐Trp_2_ or pGFP‐Trp_3_ and growing on agar fluoresced under UV irradiation. Conversely, attenuation of fluorescence was observed with pGFP‐Trp_4_, pGFP‐Trp_5_ or pGFP‐Trp_6_ (Fig. 2A). In addition, the fluorescence intensity of the cells in liquid medium decreased as the number of Trp codons increased (Fig. 2B). Fluorescence was barely observed in pGFP‐Trp_6_. To investigate the effect of hydrophobicity on fluorescence, pGFP‐Leu_*n*_ (*n* = 2, 4, 6) and pGFP‐Gly_*n*_ (*n* = 2, 4, 6) were constructed. The hydrophobicity scales of Trp, Leu and Gly are −3.4, −1.8 and 0.0, respectively 12. Fluorescence was observed in *E. coli* harboring all these plasmids, except for pGFP‐Leu_6_ (Fig. 2C,D). Thus, fluorescence of GFP was attenuated as the number of hydrophobic acid residues at the C terminus increased, and more than five hydrophobic amino acids extinguished the fluorescence almost entirely. This result is consistent with our previous hypothesis that the fluorescence intensity of GFP‐fused polypeptides decreases with the product of the average hydrophilicity of the fused segment multiplied by the number of fused amino acids 5, since we also suggested that this rule could be applied not only to peptides but also to small numbers of amino acid residues.

### Factors decreasing fluorescence intensity

**Figure 3:**
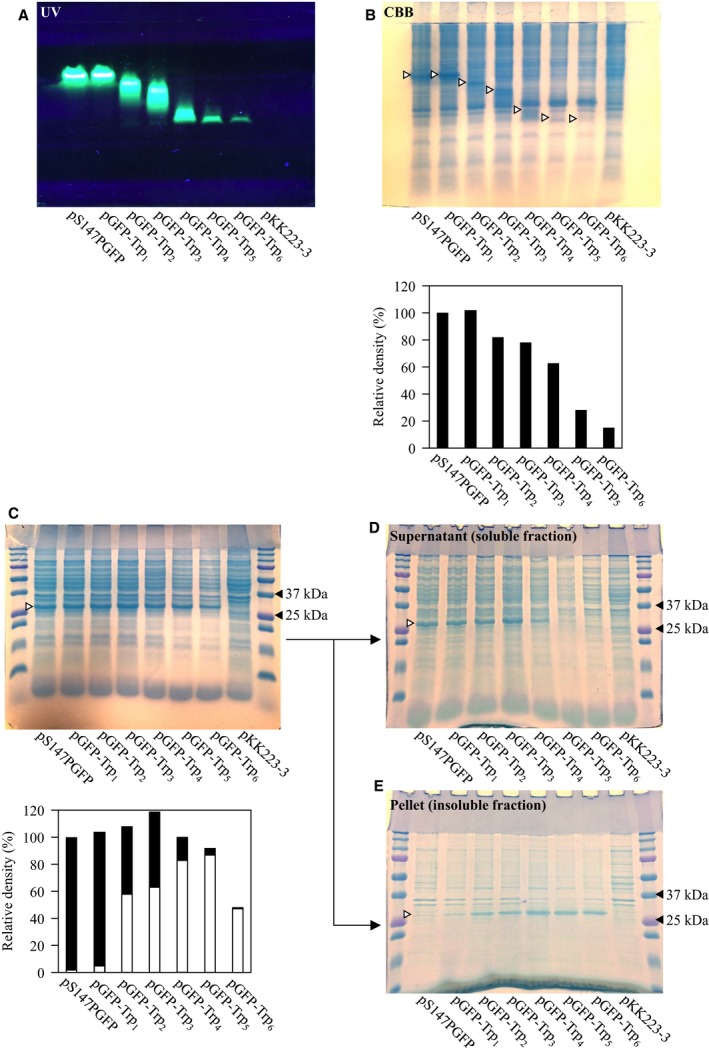
SDS/PAGE of sonicates of *Escherichia coli* harboring pGFP‐Trp_*n*_ detected using UV irradiation (A) or CBB staining (B). The open triangles in (B) show the positions of the fluorescing bands in (A). The bar graph in the lower part of (B) shows the relative densities of the CBB‐stained bands indicated by open triangles. Relative densities are shown with the band density of pS147PGFP set to 100%. SDS/PAGE of boiled sonicate of *E. coli* harboring pGFP‐Trp_*n*_ (C). SDS/PAGE of boiled supernatant (D) and pellet (E) of sonicate of *E. coli* harboring pGFP‐Trp_*n*_. The open triangles in (C–E) show the position of GFP (approximately 27 kDa). The stacked bar graph in the lower part of (C) shows the relative density of the 27 kDa bands in the upper part of (C). The density of pS147PGFP was set as 100%. The closed bar and open bar indicate the apparent abundance of GFP‐Trp_*n*_ in the supernatant and pellet, respectively.

We considered the following factors in the loss of fluorescence in *E. coli* harboring pGFP‐Trp_6_: (a) the codons added at 3′ end of *GFP* decrease translation efficiency, (b) Trp residues inhibit the folding of GFP, and (c) the residues attenuate fluorescence by acting as a ‘quencher’ that absorbs excitatory or emitted light. To investigate hypothesis (a), the CTG codons of Leu of pGFP‐Leu_*n*_ (*n* = 2, 4, 6) were replaced with TTA, since Trp has no synonymous codons, and the fluorescence intensities of *E. coli* harboring these plasmids were measured. The codon usages of CTG and TTA are 46.9‰ and 15.2‰, respectively 13. If translation efficiency and the fluorescence intensity were related to each other, then the fluorescence intensity of the *E. coli* harboring pGFP‐Leu(CTG)_*n*_ would be expected to be higher than that of pGFP‐Leu(TTA)_*n*_. However, there was no significant difference between them (Fig. 2C,D). Therefore, it appeared that the codons added at the 3′ end of *GFP* did not markedly affect its expression. To investigate hypothesis (b), sonicates of *E. coli* harboring pGFP‐Trp_*n*_ were analysed by SDS/PAGE. GFP is stable in 1% SDS, and its migration can be traced in real time using UV illumination 14. In native SDS/PAGE, fluorescence was attenuated, and the migration distance of the fluorescing band increased with the number of Trp residues (Fig. 3A). The increased migration may have been due to the binding of more SDS to Trp residues as the Trp chain was extended in the different constructs. In addition, the attenuation of fluorescence associated with Trp extension corresponded with the staining intensity of CBB (Fig. 3B). However, in denaturing SDS/PAGE, GFP‐Trp_*n*_ bands were observed close to 25 kDa (the molecular mass of GFP is 27 kDa), regardless of the number of Trps added (Fig. 3C). These results indicate that non‐fluorescent GFP migrates at a different position to fluorescent GFP (Fig. 3A,B). The differing migration positions suggest that a structural change had occurred in non‐fluorescent GFP from the addition of Trps at the C terminus. To investigate this structural change in detail, the sonicate was separated into supernatant (soluble fraction) and pellet (insoluble fraction) by centrifugation and each boiled product was subjected to SDS/PAGE. Bands of GFP containing added Trp were observed in both the soluble and insoluble fraction (Fig. 3D), with those containing five or six added Trps being more abundant in the insoluble material (Fig. 3E). These results suggest that multiple Trps at the C terminus sterically inhibit correct folding of the GFP and result in insolubility. Therefore, it seemed likely that the additional Trps did not reduce fluorescence because of quenching [hypothesis (c)]. Many examples have been reported in which the addition of a peptide, His‐tag or heterologous protein negatively affects the solubility of the target protein of interest 15, 16, 17, 18, 19. The C‐terminal Trp chain may have a negative effect on the formation of the 11th strand of GFP, which is essential for fluorescence 20. However, we did not attempt to clarify the detailed mechanism by which multiple C‐terminal Trps reduce GFP solubility in this study. This will be addressed in a future experiment appending Trp chains to a robustly folded (superfolder) GFP 19 or self‐assembly experiments with the 1–10th strands of GFP and 11th strand appending the Trp chain 20.

### Application of pGFP‐Trp_6_ as a cloning vector

**Figure 4:**
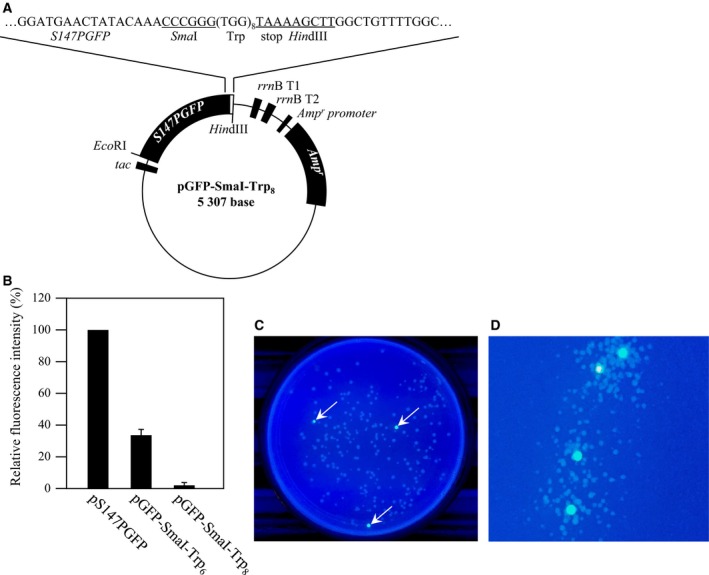
Structure of pGFP‐SmaI‐Trp_*n*_ (A), fluorescence intensity of *Escherichia coli* harboring pGFP‐SmaI‐Trp_*n*_ (B) and screening of recombinant *E. coli* using pGFP‐SmaI‐Trp_8_ (C, D).

If the multiple Trp codons of pGFP‐Trp_6_ are not expressed, the resulting GFP will fluoresce. Based on this principle, we constructed a cloning vector that can be used to identify recombinant *E. coli* colonies by GFP fluorescence. This vector has an *Sma*I recognition site between the *GFP* and Trp codons. So if a DNA fragment with a stop codon at the 5′‐end is inserted in the *Sma*I site, recombinant *E. coli* colonies will fluoresce upon UV irradiation, making it easy to screen for recombinant. First, we constructed pGFP‐SmaI‐Trp_6_ (Fig. 4A). However, *E. coli* harboring this construct fluoresced slightly; this small recovery of fluorescence may have been due to two amino acid residues (Gly‐Pro) being inserted at the *Sma*I recognition site (GGGCCC). We therefore constructed pGFP‐SmaI‐Trp_8_, to offset the effect of the Gly‐Pro residue, and *E. coli* harboring this construct did not fluoresce (Fig. 4B). After digestion of pGFP‐SmaI‐Trp_8_ using *Sma*I, a DNA that had been amplified by PCR using a primer having a stop codon at the 5′‐end was inserted into the plasmid. The recombinant *E. coli* colonies fluoresced intensely (Fig. 4C). Compared with blue/white selection, the screening for recombinants using fluorescent protein as an indicator does not require isopropyl‐β‐d‐thiogalactopyranoside or 5‐bromo‐4‐chloro‐3‐indolyl‐β‐d‐galactoside and is easy to perform 21, 22. ‘Turn‐on’ vectors (where recombinants acquire fluorescence) are superior to ‘turn‐off’ vectors (where recombinants lose fluorescence), because recombinant colonies that emit fluorescence are easier to identify than ones that do not 22. Moreover, even if non‐recombinant satellite colonies grow, it is still possible to select recombinants only (Fig. 4D). We have not yet determined the frequency of false positives, so we cannot decisively conclude that it is low. However, when this plasmid was cleaved with *Sma*I and subjected to self‐ligation using T4 DNA ligase, fluorescent colonies appeared at a frequency of only 0–1 per 50–100 colonies (data not shown). The ‘turn‐on’ vector that we reported previously was fortuitously obtained by random mutation 22. However, the ‘turn‐on’ vector developed in this study was intentionally designed based on our finding from the previous ‘turn‐on’ vector that the addition of hydrophobic peptides to GFP attenuates its fluorescence. As long as the positional relationship between GFP and the Trp chain is retained, transformation efficiency of *E. coli*, ligation efficiency and the false positive (or negative) rate might be improved by changing the plasmid size and/or cloning site.

## Conclusion

A tandem repeat of six Trps at the C terminus of GFP prevents its folding and markedly decreases its fluorescence intensity. Using this phenomenon, we constructed a cloning vector that facilitates identification of recombinant colonies of *E*.* coli* by activation of GFP.

## Author contributions

ST designed and performed all experiments. TK and RK prepared recombinants. SI and IT performed electrophoresis. NH and TA supervised the study. ST wrote the manuscript.
